# A temporal analysis on patient and health service delays in pulmonary tuberculosis in Portugal: inter and intra‑regional differences and in(equalities) between gender and age

**DOI:** 10.1186/s12889-022-14216-3

**Published:** 2022-09-28

**Authors:** Bhaswar Chakma, Dulce Gomes, Patrícia A. Filipe, Patrícia Soares, Bruno de Sousa, Carla Nunes

**Affiliations:** 1 Research Centre for Mathematics and Applications, Institute for Advanced Studies and Research, University of Évora, Évora, Portugal; 2 Department of Mathematics, School of Science and Technology, University of Évora, Évora, Portugal; 3 Quantitative Methods for Management and Economics Department, Iscte Business School, Iscte - University Institute of Lisbon, Lisboa, Portugal; 4 Public Health Research Centre, NOVA National School of Public Health, Universidade NOVA de Lisboa, Lisboa, Portugal; 5 Comprehensive Health Research Center, Universidade NOVA de Lisboa, Lisboa, Portugal; 6 CINEICC, Faculty of Psychology and Educational Sciences, University of Coimbra, Coimbra, Portugal

**Keywords:** Tuberculosis, Delay, Portugal, ARIMA, Temporal trends

## Abstract

**Background:**

Tuberculosis (TB) diagnosis and treatment delays increase the period of infectiousness, making TB control difficult and increasing the fatality rates. This study aimed to determine the evolution of health care service delay (time between the patient’s first contact with the health service and the diagnosis/start of treatment) and patient delay (time between onset symptoms date and the date of first contact with health services) for Pulmonary Tuberculosis (PTB) in Portugal between 2008 and 2017 across different regions, age groups and gender.

**Methods:**

An exploratory analysis was performed, trends of both delays were studied, and 36 months forecasts were generated. We used the permutation test to test differences between groups and the Seasonal and Trend decomposition using Loess (STL) method and Autoregressive Integrated Moving Average (ARIMA) models for forecasting for both Health and Patient delays. We used data from notified PTB cases in mainland Portugal between 2008 and 2017, provided by the national surveillance system.

**Results:**

Health delays remained relatively constant while patient delays increased. Females had significantly higher health delays in some regions. Individuals older than 64 had higher health delays than younger individuals, while patient delay for working-age individuals between 15 and 64 years old, presents higher patient delay.

**Conclusions:**

Forecasts presage that the upward trend of the delays is unlikely to fall in the coming years. It is important to understand the evolution of the delays and predict how these will evolve. Our understanding of the delays behaviours will contribute to better health policies and resources allocation.

## Background

Tuberculosis (TB), a lethal infectious disease, is a global health problem. According to a recent report by the World Health Organization (WHO), the disease – ranking above HIV/AIDS – is one of the top 10 causes of death worldwide [1]. In 2019, WHO reported that an estimated 10 million people became infected with TB, and of those infected, 2.5% were from Europe. Although the number of infected people is low in Europe, in 2018, Portugal had a notification rate of 20.8 per 100,000 population, ranking 3^rd^ in the European Union/the European Economic Area [2].

TB diagnosis and treatment delays, which could be due to health care services or patients or both, increase the period of infectiousness [3–6]. Such delays make TB control difficult and increase fatality rates. Several studies on delays were focused on African and Asian countries, where TB infection rates are higher, and where cultural beliefs and health systems are different [7, 8]. There exist many studies worldwide on delay diagnosis and treatment of TB, using a temporal approaches. However, in European countries there are few and, in particular, we must highlight that this study is the first to apply this type of temporal approach to both patient and health delays for PTB data in Portugal. In particular, to the best of our knowledge, only [9–14] studied PTB diagnosis and treatment delays in Portugal. In those studies, the authors investigated factors related to the delays using different statistical methodologies, such as survival analysis, structured additive regression models, or generalized linear models. None of the studies analyzed the delays and their patterns in time series context, nor how the delay varied across the different regions. The use of time series techniques enables identifying the evolution patterns of the delays over time and predicting what will happen in the future, assuming the same patterns continue to hold. In particular, these techniques allow us to conduct trend analysis, which are extremely useful in making informed decisions for the future and comparing regional symmetries/asymmetries with the delays, which is helpful in heterogeneous diseases.

Countries with low TB incidence usually have a heterogeneous geographical distribution of the disease, making Portugal an excellent case study for TB diagnosis delay. TB incidence rate is higher, above 20 per 100,000 inhabitants, in urban regions such as the North and Lisbon and Tagus Valley (LTV). In contrast, the Center and Alentejo are regions with lower incidence, with a higher proportion of older individuals [15]. Thus, by studying delays across different geographical regions, one can understand which regions need immediate action and tailor different interventions according to the characteristics of the regions. Portugal has five Regional Health Administrations (North, Centre, Lisbon and Tagus Valley, Alentejo and Algarve) and the two Regional Health Secretariats from the Autonomous Regions Islands of Azores and Madeira, not included in this study. The report [15] includes information on how the Portugal regions are progressing in aspects related to socio-economic characteristics, such as economic development, health, well-being and others. The aim of this study is to determine the evolution of both health care service delays and patient delays of PTB in Portugal, between 2008 and 2017, across the different regions, age groups and gender.

## Methods

### Data

We used data from the national surveillance system for TB (SVIG-TB), which includes those diagnosed with pulmonary and extrapulmonary tuberculosis. We have only worked with pulmonary tuberculosis (PTB) cases notified in Portugal from January 2008 to December 2017. In our study we considered the data of mainland Portugal, comprised of five regions: North, Center, Lisbon and Tagus Valley, Alentejo and Algarve. We analyzed two types of delays: Patient Delay and health service delay, now referred as Health Delay, considering region, age and gender of the patient. Health delay was defined as the time between the patient’s first contact with the health service and the diagnosis/start of treatment. Patient delay was defined as the time between onset symptoms date and the date of first contact with health services. Delays were measured in days and age was further categorised into four groups: 0-14, 15–44, 45–64, and>64 years.

We dropped observations that had one of the three anomalies: (1) Inconsistencies with (i) symptoms onset and/or first appointment or with (ii) symptoms onset and microscopy and/or culture; (2) Contact screening and symptoms onset after appointment; (3) Same date for symptoms onset, appointment, and diagnosis. We also dropped observations that had delays over one year. In addition, since we analyzed the delays by different categories, we dropped missing cases on an analysis-by-analysis basis.

### Methods

Time series data can be split into many components, each representing an underlying pattern category. A commonly used decomposition method is STL, an acronym for Seasonal and Trend decomposition using Loess. This robust and versatile decomposition method was developed by [16]. The STL procedure employs a succession of Loess smoothers, which consist of locally weighted polynomial regressions at each point of the series, in which the explanatory variables are the closest values to this response value to be estimated [17].

Another standard modelling tool in time series analysis is the non-seasonal ARIMA, which combines the ARMA model with differencing and can be used when data is non-stationary. A stationary series is roughly horizontal with constant variance and cannot be predicted in the long-term. To make a non-stationary series stationary, differencing or transformation or both are required. Differencing helps to stabilize the mean; Box-Cox transformation stabilizes the variance [18].

These models are expressed as ARIMA$(p,d,q)$, where $p =$ order of the autoregressive part (lagged observations as inputs); $d =$ degree of first differencing involved; $q =$ order of the moving average part (lagged errors as inputs), and can be written as

$$\begin{aligned} (1-\phi _1B-\cdots -\phi _pB^p)(1-B)^d y_ t= c+ (1+\theta _1B+\cdots +\theta _qB^q)\varepsilon _{t} \end{aligned}$$

where $\varepsilon _t,\, t\in Z$, is white noise. This is a multiple regression with lagged values of $y_t$ as predictors.

Seasonal ARIMA models are an extension of non-seasonal models. It incorporates the seasonality of data in the model, which could be expressed as ARIMA (*p*, *d*, *q*) $(P, D, Q)_{S}$, where $S =$ period of seasonality. The parameters of the ARIMA models are estimated by maximum likelihood estimator. For further documentation on the theoretical discussion see [19].

In this study, Patient Delay and Health Delay trend were estimated using Seasonal-trend LOESS (STL) decomposition, and autoregressive integrated moving average (ARIMA) was used to generate trend forecasts. Additionally, we also employed a permutation test to determine whether the differences found in the means and medians delays between gender and age groups were statistically significant. The advantage of using permutation tests rely on the absence of a distributional assumption on the two populations under consideration.

We used the statistical software R 4.0.3 [20] to conduct the analysis, particularly, the fable package to build ARIMA models [21]. For further documentation on practical implementation in R see [22], and [21] for detailed instructions on fable.

## Results

### Characterization of the delays

**Fig. 1:**
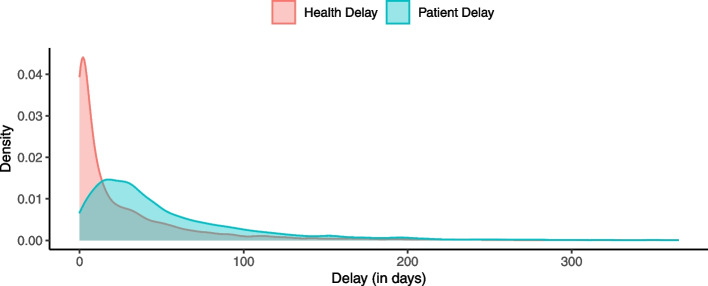
Mainland Portugal Delays: Density Plot

Figure 1 presents density plots of health and patient delays. Clearly, for both type of delays, the distribution is right skewed. This fact is also reflected in the summary statistics tables; see Tables 1 and 2. Note that in our final data set the minimum delay is 0 day and maximum delay is 365 days. Since these values are fixed, we do not report them in the tables.

**Table 1:** Health Delay: Total number of cases, number (%) of dropped (drop.) cases, final sample size, statistics for the delay in days - 25% and 75% percentiles, mean, median and standard deviation (Mainland Portugal (PT), by region, by gender and by age category)

	cases	drop.	% drop.	n	p25	p75	mean	median	sd
**Mainland PT**	15,084	3,654	24.2	11,430	2	36.0	28.5	10.0	45.7
**Region**									
Alentejo	537	136	25.3	401	2	51.0	35.4	14.0	48.8
Algarve	776	321	41.4	455	3	31.5	24.4	10.0	36.7
Center	1,400	452	32.3	948	1	36.0	26.6	8.0	41.6
LTV	5,918	1,399	23.6	4,519	2	38.0	30.2	10.0	49.3
North	6,453	1,346	20.9	5,107	2	34.0	27.3	10.0	43.5
**Gender**									
Male	10,574	2,518	23.8	8,056	1	33.0	26.3	9.0	43.2
Female	4,510	1,136	25.2	3,374	3	43.0	33.8	13.0	50.9
**Age**									
0-14	171	41	24.0	130	3	28.0	26.0	8.5	44.1
15-44	7,114	1,573	22.1	5,541	1	28.0	23.6	7.0	41.1
45-64	5,029	1,247	24.8	3,782	2	35.0	28.5	9.5	46.4
> 64	2,754	786	28.5	1,968	5	58.3	42.7	25.0	53.3
Unknown	16	7	43.8	9	2	23.0	12.6	8.0	13.3

**Table 2:** Patient Delay: Total number of cases, number (%) of dropped (drop.) cases, final sample size, statistics for the delay in days - 25% and 75% percentiles, mean, median and standard deviation (Mainland Portugal (PT), by region, by gender and by age category)

	cases	drop.	% drop.	n	p25	p75	mean	median	sd
**Mainland PT**	15,084	4,659	30.9	10,425	18.0	69.0	52.9	36.0	52.9
**Region**									
Alentejo	537	159	29.61	378	15.3	65.8	50.5	31.0	54.8
Algarve	776	339	43.7	437	17.0	60.0	47.1	29.0	51.3
Center	1,400	534	38.1	866	14.0	58.8	46.1	29.0	50.9
LTV	5,918	1,748	29.5	4,170	21.0	76.0	57.0	40.0	53.3
North	6,453	1,879	29.1	4,574	17.0	65.0	51.2	35.0	52.6
**Gender**									
Male	10,574	3,241	30.7	7,333	18.0	70.0	53.1	36.0	52.5
Female	4,510	1,418	31.4	3,092	18.0	69.0	52.6	34.5	53.8
**Age**									
0-14	171	58	33.9	113	16.0	56.0	41.2	26.0	39.0
15-44	7,114	2,006	28.2	5,108	19.0	70.0	53.1	37.0	50.7
45-64	5,029	1,600	31.8	3,429	19.0	74.0	56.8	39.0	56.4
> 64	2,754	986	35.8	1,768	14.0	57.0	45.8	29.0	52.1
Unknown	16	9	56.3	7	17.0	33.5	25.1	26.0	11.5

**Fig. 2:**
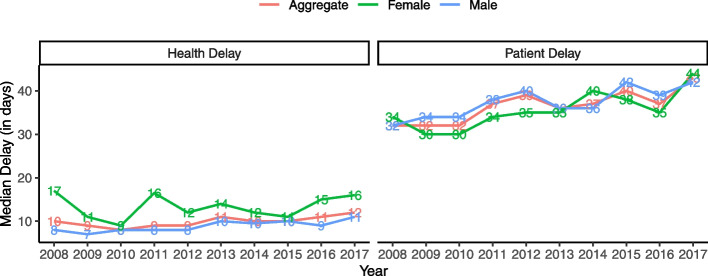
Median health and patient delay by gender in mainland Portugal

Figure 2 presents the median patient and health delay in mainland Portugal and the combination of both delays, named Aggregate, between 2008 and 2017. Additionally, the figures also display the median delays by gender over time. Overall, patient delays are higher than health delays. Both delays seem to present a slightly increasing trend over time, especially for the patient delay. Health delay was consistently higher for females than males, though the difference is minimal in some years.

**Fig. 3:**
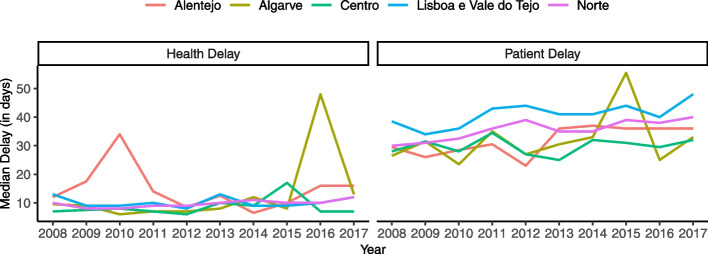
Median health and patient delay by region in mainland Portugal

**Fig. 4:**
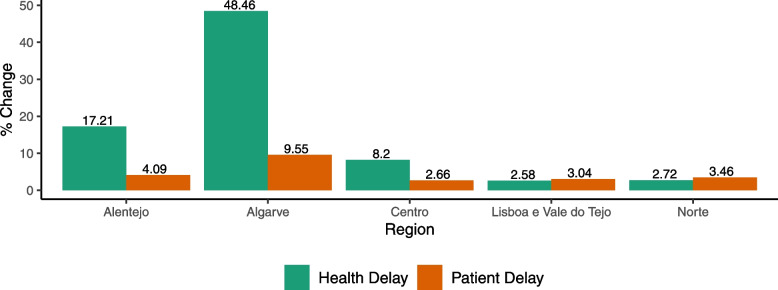
Average yearly changes in patient and health delays for each region

Figure 3 presents the median health and patient delay by region. Overall, patient delay seems to be increasing over time. Patient delay was consistently higher in Lisbon and Tagus Valley than the remaining regions under analysis, except in 2015 when Algarve had the highest patient delay of all regions. Health delay seems to remain relatively constant over time and similar between regions, except in 2010 and 2016 when Alentejo and Algarve, respectively, had higher health delays than the remaining regions. These spikes are evident when comparing the average annual changes in mean delay for each region, displayed in Fig. 4.

**Fig. 5:**
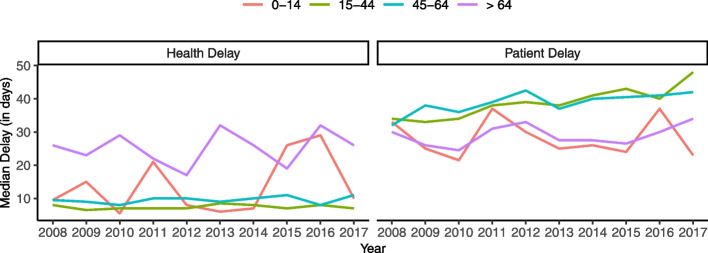
Median health and patient delay by age group in mainland Portugal

Figure 5 shows the annual median patient and health delays by age group. Overall, health delay remains slightly constant over time. Individuals above 64 years old had higher health delays than the remaining age groups over time, while individuals younger than 15 displayed an irregular trend for health delay, even surpassing the older group in 2015. On the contrary, analysing patient delay, these two groups of individuals (between 0 and 14 years old and older than 64) had the smaller patient delay. Overall, patient delay seems to be slightly increasing over time.

**Fig. 6:**
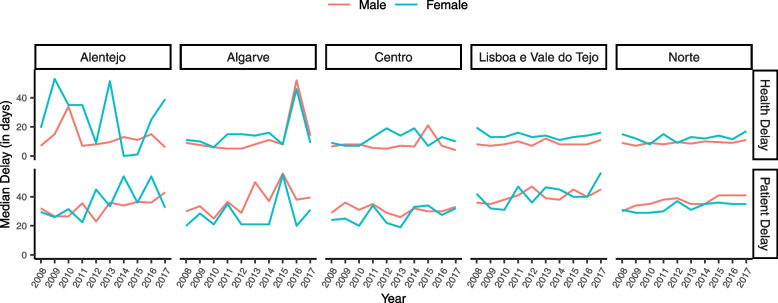
Median health and patient delay by region and gender in mainland Portugal

**Fig. 7:**
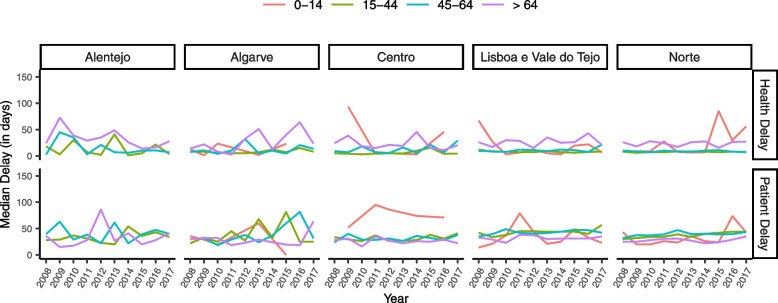
Median health and patient delay by region and age group in mainland Portugal

Figure 6 presents the yearly patient and health median delay for each region and gender. Alentejo presents the most irregular health and patient delay trends, likely due to fewer individuals in this region. For most of the years under analysis, females have had higher health delays than males for all the regions. Figure 7 exhibits the regional yearly patient and health median delays by age group. Overall, the variation in patient delay between age groups was minimal, except in Center, where individuals older than 64 had higher patient delay than the remaining age groups.

**Fig. 8:**
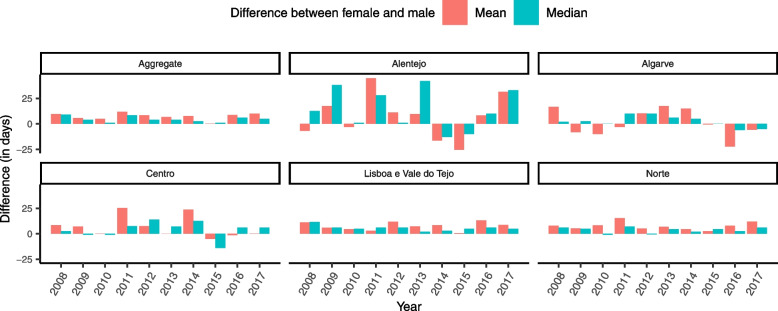
Difference between male and female health delays for each region in mainland Portugal

Figure 8 shows the differences in yearly mean and median health delays between females and males across regions. Females had higher delays than males in most years across regions. In particular, differences were consistently positive in Lisbon and Tagus Valley.

To ascertain whether the differences found between genders was statistically significant, we conducted permutation tests. Our null hypothesis was the non-existence of differences in health delay between males and females, whereas our alternative hypothesis stated that females had higher health delays than males. We resampled each region 1,000,000 times and conducted permutation tests. Table 3 presents the observed differences and the corresponding *p*-values. Health delay was statistically different between genders for all regions, except for Algarve.

**Table 3:** Results of the permutation test for gender differences in health delay

	Observed Difference		*P*-Value	
Region (Observation)	Mean	Median	Mean	Median
Alentejo (Female = 97; Male = 304)	8.53	13	0.070	0.018
Algarve (Female = 111; Male = 344)	2.72	3	0.245	0.179
Center (Female = 281; Male = 667)	6.73	4	0.013	0.080
LTV (Female = 1504; Male = 3015)	7.55	6	<0.001	<0.001
North (Female = 1381; Male = 3726)	7.70	3	<0.001	0.001

Note: For each region the number of resamples is 1,000,000

We also tested whether individuals older than 64 had higher health delays than younger individuals ($H_0$: health delays are not different between individuals >64 vs $\le$64 *vs* $H_1$: individuals >64 have higher health delays than individuals $\le$64.). We conducted permutation tests with 1,000,000 resamples for each region. Table 4 presents the observed differences and the corresponding *p*-values. The results indicate statistically significant differences, for all regions, in health delays between individuals older than 64 years of age and younger. Similar tests were made for patient delays, but in this case there were no differences detected.

**Table 4:** Results of the permutation test for differences between individuals >64 vs $\le$64 in health delay

	Observed Difference		*P*-Value	
Region (Observation)	Mean	Median	Mean	Median
Alentejo (>64 = 118; $\le$64 = 283)	20.26	26.5	<0.001	<0.001
Algarve (>64 = 96; $\le$64 = 359)	23.69	18.5	<0.001	<0.001
Center (>64 = 185; $\le$64 = 763)	9.92	14.0	0.003	<0.001
LTV (>64 = 656; $\le$64 = 3,855)	20.31	18.0	<0.001	<0.001
North (>64 = 913; $\le$64 = 4,193)	15.35	16.0	<0.001	<0.001

Note: For each region the number of resamples is 1,000,000

### Temporal analysis

We used ARIMA models to forecast trend components of monthly health and patient delays in mainland Portugal. To obtain trend component, we used seasonal window of 13 and trend window of 21 in STL decomposition. Although there is no strict rule for these parameters, the setting we used gives a good balance between overfitting the seasonality and allowing it to change over time slowly [22]. We fitted several models and evaluated the accuracy of their forecast using a train-test setting (training data: 2008-2014; test data: 2015-2017). Table 5 presents the final top three models based on the lowest root mean squared scaled error (RMSE), the mean average error (MAE), the mean average percentage error (MAPE) and mean absolute scaled error (MASE) are also shown.

**Table 5:** Results of the top three ARIMA models: Point forecast accuracy

	Model	RMSE	MAE	MAPE	MASE
**Health Delay**					
	ARIMA(2,1,1)(2,0,0)[12] with constant	1.0158	0.8587	7.8088	0.7853
	ARIMA(1,1,0)(2,0,0)[12] with constant	1.0206	0.8656	7.8827	0.7916
	ARIMA(1,1,0)(2,0,0)[12]	1.0232	0.8674	7.8958	0.7933
**Patient Delay**					
	ARIMA(4,1,0)(0,0,1)[12]	1.4775	1.2136	3.1758	0.6674
	ARIMA(4,1,0)(1,0,0)[12]	1.5664	1.3344	3.5031	0.7339
	ARIMA(1,1,3)(0,0,1)[12]	2.3132	2.0653	5.4028	1.1358

**Fig. 9:**
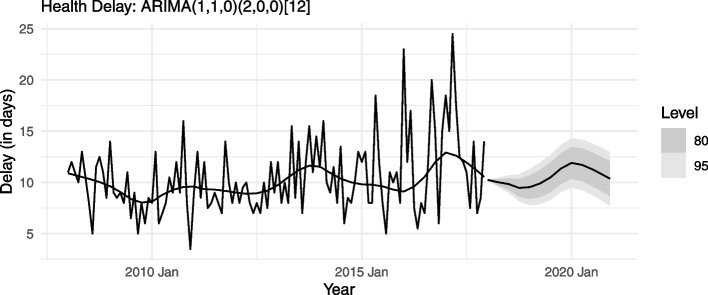
Forecast (Health Delay): Trend component of monthly median

**Fig. 10:**
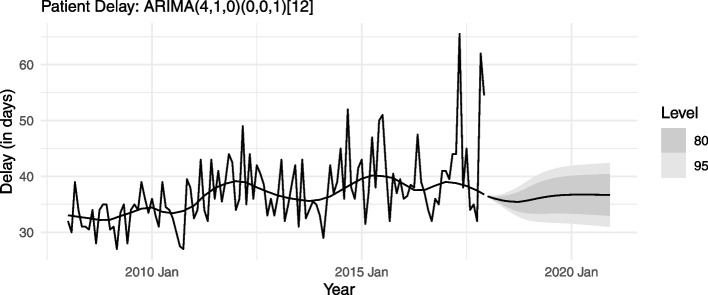
Forecast (Patient Delay): Trend component of monthly median

For each delay type (health and patient), all the three models – with negligible differences in RMSEs – yield very similar forecasts. Figures 9 and 10, taking the parsimonious model for each delay type, present the trend components’ forecasts for the next 36 months (2018-2020). These forecasts indicate that both health delay and patient delay in mainland Portugal are unlikely to fall in the coming years.

## Discussion

Between 2008 and 2017, we found that health delay remained relatively constant in mainland Portugal, while patient delay increased. Overall, regions maintained the same trend over the years, except for Alentejo and Algarve, with higher yearly variations. Females had significantly higher health delays than males in some areas of the country. This pattern was not as evident for the patient delay. Similarly, we found that individuals older than 64 had higher health delays than younger individuals across mainland Portugal. Patient delay displayed an opposite trend, with working-age individuals, between 15 and 64 years old, presenting higher patient delay. The forecasts estimate a similar trend for the next years.

In 2017, we found a median patient delay of 43 days and a health delay of 12 days in mainland Portugal. Although there is no universal consensus on the acceptable time between symptoms onset and TB diagnosis, several authors used cut-offs to define diagnosis delay. For health delay, the cut-off ranged from 14 to 21 days, while patient delay had a range of 10 to 35 days [23, 24]. Although our median health delay was 12 days and deemed acceptable by several authors, the percentile 75 for mainland Portugal was 36 days. [9], in a study conducted in Portugal which included pulmonary and extrapulmonary TB, also found a smaller median patient delay and higher median health delay, 33 and 17 days, respectively [9]. However, a systematic review in 2008, including high and low-burden countries, found no pattern regarding patient and health delay contributions to the total delay [25].

We also compared the health delay between males and females and individuals older and younger than 64. The literature seems to indicate that older age is associated with higher delays [26, 27]. However, other studies found no association, which could be due to different classifications of age. Some considered age as above 60, 70, or distinct age groups [9, 23, 26, 27]. We found that females had significantly higher health delays than males in most regions, which was replicated in other studies [23, 25–27]. However, this finding is also inconsistent across the literature [9, 25] and several studies also include extrapulmonary TB. Regional differences might also explain some differences obtained and could be responsible for some of the inconsistencies found in the literature. Several factors were associated with higher delays, such as living in a rural area [23, 25, 28], not having information about TB, low health literacy [28, 29], living more than 10km away from the nearest health facility [28], living far from pharmacies [30], and seeking public low-level healthcare facilities first [25]. All these factors differ geographically and between regions. Thus, gender and age might be confounding factors for some of the characteristics of the region. Further studies should explore how different characteristics of the regions, such as access to health facilities, number of doctors available, health literacy, and residency, affect TB diagnosis delays and understand the true effect of gender and age.

We did not study the factors associated with patient and health delay as our aim was to determine the evolution of both delays across the different regions, age groups and gender. Some of our results should be further explored, namely the gender and age difference in health delays. We used the national TB surveillance database that does not collect such detailed information about the region. There is no information regarding distance to the nearest health center or health facilities, and it is unclear which health facility the patient first went. Further studies should explore whether females have higher health delays or if gender is confounding for other characteristics.

Our study has some limitations common on this type of studies. Regional delays were highly variable, primarily due to the low number of yearly diagnosed individuals in some regions. Our study also suffers from recall bias, as the patient has to rely on their memory to identify symptoms onset, which might even be confounding in the case of comorbidities with similar symptoms. It is also of extreme importance to consider some possible limitations and biases that can appear when dealing with the SVIG-TB database. Although it was not designed for research, the available information is consistent and reliable when compared to studies of tuberculosis surveillance and monitoring in Europe from entities such as WHO. Worth noting is that levels of missingness in our database is about 24% for patient delay and 31% for health delay. Additionally, health delays might also be biased. Health delay was defined as the time between the patient’s first contact with the health service and the diagnosis/start of treatment. However, this time does not account for possible delays between the schedule and the realization of the consultation. In regions with fewer doctors or health facilities, this time might be higher and incorrectly increase patient delay instead.

## Conclusions

In our study we analysed all PTB cases notified between 2008 and 2017 in mainland Portugal, which allowed us to understand the evolution pattern of health and patient delay across different regions, gender and age groups. We have observed that health delays remained relatively constant in mainland Portugal while patient delays increased. Significant differences were detect between gender, where females had significantly higher health delays than males in some regions of the country. Individuals older than 64 years old had higher health delays than younger individuals across mainland Portugal. In terms of prediction, we foresee that the upward trend of the delays is unlikely to fall in the next years.

TB diagnosis delays threaten TB control, especially in high-income countries that have lower TB incidence rates. However, the COVID-19 pandemic poses a new threat. Although, at the moment it is still unclear the effect of the pandemic in TB diagnosis and delays [31] compared individuals diagnosed with TB in 2019 and 2020 during the pandemic and found a reduction in TB diagnoses and an increase in the median patient delay (2019: 30 days vs 2020: 75 days). However, further evidence is needed to understand the impact of the pandemic in TB. It is essential to understand the evolution of the delays and predict how these will evolve. Our understanding of the delays behaviours will contribute to better health policies and resources allocation.

## Data Availability

The dataset supporting the conclusions of this article was obtained through the Portuguese National Tuberculosis Surveillance System (SVIG-TB) and is not publicly available. The SVIG-TB is supervised by the Directorate Gen- eral of Health (Direção Geral da Saúde, DGS) that grants access to the fully anonymized dataset for epidemiological studies. The aggregated dataset used in this specific study is available from the corresponding author upon reasonable request.
