# Correction to: a temporal analysis on patient and health service delays in pulmonary tuberculosis in Portugal: inter and intra‑regional differences and in(equalities) between gender and age

**DOI:** 10.1186/s12889-023-15406-3

**Published:** 2023-03-14

**Authors:** Bhaswar Chakma, Dulce Gomes, Patrícia A. Filipe, Patrícia Soares, Bruno de Sousa, Carla Nunes

**Affiliations:** 1 Research Centre for Mathematics and Applications, Institute for Advanced Studies and Research, University of Évora, Évora, Portugal; 2 Department of Mathematics, School of Science and Technology, University of Évora, Évora, Portugal; 3 Quantitative Methods for Management and Economics Department, Iscte Business School, Iscte – University Institute of Lisbon, Lisboa, Portugal; 4 Public Health Research Centre, NOVA National School of Public Health, Universidade NOVA de Lisboa, Lisboa, Portugal; 5 Comprehensive Health Research Center, Universidade NOVA de Lisboa, Lisboa, Portugal; 6 CINEICC, Faculty of Psychology and Educational Sciences, University of Coimbra, Coimbra, Portugal

**BMC Public Health (2022) 22:1830**

10.1186/s12889-022-14216-3

The original publication of this article contained an incomplete funding section. The complete funding section is shown in this correction article, the original article has been updated.

Funding

Fundação para a Ciência e Tecnologia provided financial support for the conduct of this research [Grants: PTDC/SAU-PUB/31,346/2017-UrbanTB: From symptoms to diagnosis of Urban Tuberculosis, considering individual and contextual factors. What are the determinants and critical points of this delay’s pathway? and UID/MAT/04674/2020], but has had no involvement in the research itself.

